# Detection of KRAS, NRAS and BRAF by mass spectrometry - a sensitive, reliable, fast and cost-effective technique

**DOI:** 10.1186/s13000-015-0364-3

**Published:** 2015-07-30

**Authors:** Mark Kriegsmann, Norbert Arens, Volker Endris, Wilko Weichert, Jörg Kriegsmann

**Affiliations:** Institute of Pathology, University of Heidelberg, INF 224, Heidelberg, Germany; Institute of Molecular Pathology, Trier, Germany; MVZ for Histology, Cytology and Molecular Diagnostics, Trier, Germany; National Center of Tumor Diseases, Heidelberg, Germany; German Cancer Consortium (DKTK), Heidelberg, Germany

## Abstract

**Background:**

According to current clinical guidelines mutational analysis for *KRAS* and *NRAS* is recommended prior to EGFR-directed therapy of colorectal cancer (CRC) in the metastatic setting. Therefore, reliable, fast, sensitive and cost-effective methods for routine tissue based molecular diagnostics are required that allow the assessment of the CRC mutational status in a high throughput fashion.

**Methods:**

We have developed a custom designed assay for routine mass-spectrometric (MS) (MassARRAY^®^, Agena Bioscience) analysis to test the presence/absence of 18 *KRAS*, 14 *NRAS* and 4 *BRAF* mutations. We have applied this assay to 93 samples from patients with CRC and have compared the results with Sanger sequencing and a chip hybridization assay (KRAS LCD-array Kit, Chipron). In cases with discordant results, next-generation sequencing (NGS) was performed.

**Results:**

MS detected a *KRAS* mutation in 46/93 (49 %), a *NRAS* mutation in 2/93 (2 %) and a *BRAF* mutation in 1/93 (1 %) of the cases. MS results were in agreement with results obtained by combination of the two other methods in 92 (99 %) of 93 cases.

In 1/93 (1 %) of the cases a G12V mutation has been detected by Sanger sequencing and MS, but not by the chip assay. In this case, NGS has confirmed the G12V mutation in *KRAS*.

**Conclusions:**

Mutational analysis by MS is a reliable method for routine diagnostic use, which can be easily extended for testing of additional mutations.

## Background

Colorectal carcinoma (CRC) is the third most common cancer in men and the second most common cancer in woman worldwide with approximately 694.000 deaths reported in 2012 [1]. It has long been recognized that the epidermal growth factor receptor (EGFR) pathway is frequently activated in CRC [2]. This results in promotion of tumor growth, inhibition of apoptosis, vascular proliferation, invasion, and metastasis.

Therefore, targeted therapies against EGFR such as cetuximab and panitumumab have been developed and are currently approved for the treatment of metastatic disease (mCRC), irrespective of whether applied in combination with conventional chemotherapy or as single agents.

Downstream signalling of EGFR activates *RAS*- and *RAF*-genes that are members of this pathway and can harbour oncogenic mutations in 30–60 % (*KRAS*) [3–6], 5–20 % (*BRAF*) [7–9] and 1–3 % (*NRAS*) [10, 11] of cases respectively.

It has been shown that activating mutations of *KRAS* or *NRAS* lead to a consecutive activation of the RAS-RAF pathway downstream of EGFR and consequently result in resistance to anti-EGFR therapy [12–14]. For this reason, currently the American Society of Clinical Oncology (ASCO) [15] and the European Society for Medical Oncology (ESMO) [16] recommend treatment with anti-EGFR antibodies only in mCRC patients with *RAS* wild-type tumors. This approach is reasonable since patients with mutated RAS have no benefit from this therapy. Additionally, it reduces overall treatment costs and prevents patients from unnecessary side effects. In 2009, when the recommendations were published, the definition of a *RAS* wild type tumor was based on a negative result, when the most common mutations in codon 12 and 13 (97 %) of exon 2 were analyzed. However, since recent studies suggest that resistance to anti-EGFR therapy might also be mediated by less frequent mutations of *KRAS* [10, 17–19] or *NRAS* [10, 11, 18] in codon 61 of exon 3 as well as codon 117 and 146 of exon 4 (3 %), it is now mandatory to include those mutational hotspots in the genetic testing, as well. This fact is underlined by a recent study where approximately 20 % of tumors originally classified as having no *KRAS* mutations in exon 2, harboured another mutation in one of the RAS genes [11].

As with K*RAS* mutations (especially codon 12), *BRAF* mutations have also been linked to a worse patient prognosis in CRC [8, 20]. However, it is important to note that the prognostic impact of the presence of a BRAF mutation is dependent on the microsatellite status: whereas microsatellite-stable BRAF mutated CRC are associated with a worse, microsatellite-instable BRAF mutated tumors are associated with a better prognosis than *BRAF* wild type CRC [21]. However, the presence of BRAF mutations currently is believed not to be predictive for the response to anti-EGFR therapies [22].

Furthermore, recent studies showed that acetylsalicylic acid and other nonsteroidal anti-inflammatory drugs are associated with reduced disease recurrence and improved outcome in CRC and that these benefits are limited to patients with *PIK3CA-*mutated cancers [17, 19, 23]. Also other genetic mutations that are frequently altered in CRC such as *p53* [24] or *PTEN* [25] may serve as prognostic or predictive biomarkers. While very promising, all lack clinical significance at present.

When the first anti-EGFR therapies for mCRC entered the arena, institutes of pathology expanded their expertise in molecular techniques in order to be able to evaluate the mutational status of *KRAS*, *NRAS* and *BRAF*.

Accurate mutation assessment depends on several factors such as available tissue, DNA-quality, percentage of tumor cells as well as the specificity and sensitivity of various test systems [26].

When choosing an assay for routine diagnostics, additional factors such as workload, time-to-results, hands-on time, equipment, assay costs, assay flexibility and robustness of the technique applied needed to be addressed [27].

Methods currently available for *KRAS*, *NRAS* and *BRAF* mutational analysis include Sanger sequencing, which is still regarded as the gold standard, and numerous alternatives as allele-specific PCR [28], single nucleotide primer extension assays [27, 29], pyrosequencing [30], real-time PCR [31], high resolution melting curve analysis [30, 32], amplification refractory mutation system (ARMS)-Scorpion assay [33], strip or chip assay combining PCR followed by hybridization to a *KRAS* or *NRAS*-specific probe [27, 34], next-generation sequencing (NGS) [34] and matrix assisted laser desorption/ionization mass spectrometry (MALDI MS) [17]. However, past studies with MALDI MS mainly applied genetic panels provided by the manufacturer that cover many mutations that are not recommended to assess. In consequence these panels are not cost-effective for routine use.

Therefore, we developed a MALDI MS test assay for the simultaneous detection of *KRAS*, *NRAS* and *BRAF* mutations in a routine high throughput setting and implemented an optimised workflow. We compared the data generated by this assay to data from standard RAS mutation detection methods, specifically the KRAS 1.4 LCD Array Kit (Chipron, Berlin) and Sanger Sequencing. Cases with discordant mutation results were subjected to NGS analysis to definitely clarify the mutational status.

## Methods

### Patients

93 tumor samples of patients with CRC were analysed using a custom panel of mutation assays across the *KRAS, NRAS and BRAF* oncogenes with the Agena Bioscience MALDI MS platform.

### Sample preparation and DNA extraction

Hematoxylin and Eosin (H&E)-stained slides from formalin-fixed paraffin-embedded (FFPE) tumor samples were reviewed by a pathologist and the tumor area was selected for analysis. A serial unstained tissue section was manually dissected and subjected to Tissue lysis buffer (Qiagen, Hilden, Germany). Following Proteinase-K digestion and a decrosslink-step, DNA was automatically isolated using a QiaSymphony^®^ device (Qiagen, Hilden, Germany) and subsequently quantified by OD 260 nm.

### Workflow

**Fig. 1:**
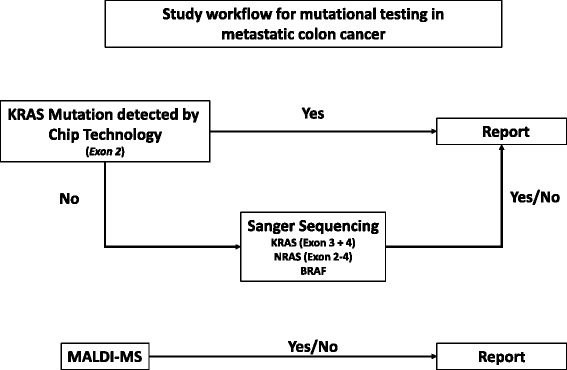
The study workflow. All cases were tested by the KRAS LCD-array Kit and MALDI MS. If a mutation was found by the KRAS LCD-array Kit, a report was signed out. If no mutation was detected by the KRAS LCD-array Kit, that covers exclusively exon 2, Sanger sequencing has been performed for *KRAS*, *NRAS* and *BRAF* in exon 2–4 and a report was signed out accordingly

A simple workflow for mutational testing has been employed in routine diagnostics, which is depicted in Fig. 1.

### KRAS LCD-Array kit

**Fig. 2:**
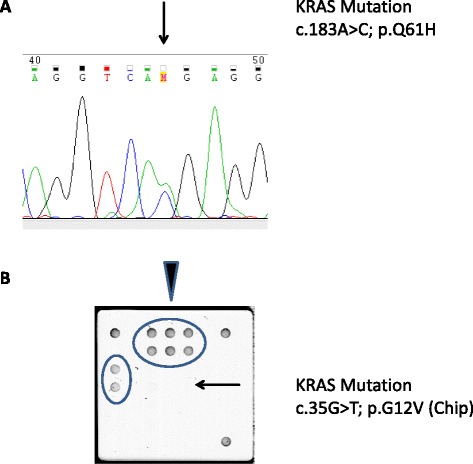
Examples of KRAS mutations. **a** A representative example of a chromatogram. A *KRAS* mutation (c.183A > C; p.Q61H) in exon 3 as detected by Sanger sequencing is depicted (arrow). Each mutation was observed on the forward and the reverse strand. **b** An example of a chip-hybridization result from one patient. On top 3 type-specific double signals (arrowhead). On the left, the mutation-specific double signal (c35G > T; p.G12V) (arrow)

The KRAS LCD-Array kit for detection of *KRAS* mutations in codon 12 and 13 (Exon 2) is based on the amplification of a short PCR fragment and the subsequent identification of point mutations by amplicon hybridization to immobilized capture probes. Biotin labelling of the generated 170 bp PCR fragment occurs during PCR amplification. Following a short hybridization to wild type and mutation specific capture probes immobilized on the surface of the LCD-Array, bound PCR fragments are visualized using the sensitive streptavidin-enzyme-substrate cascade (Fig. 2).

To detect even small amounts of mutated *KRAS* sequences within an excess amount of wild type background, amplification is carried out in the presence of the KRAS Wildtype Supressor Compound (K-RAS WSC). This molecule preferentially suppresses wild type-sequence amplification and therefore allows sequence-specific detection of smallest amounts of KRAS mutations in codon 12 and 13.

### Sanger sequencing

PCR primers were bought from Metabion (Munich, Germany). PCR amplification products were purified by ethanol precipitation and were bidirectionally sequenced using Big Dye^®^ v3.1 reagents (Thermo Fisher Scientific, Waltham, USA) according to the manufacturer’s protocol. Sequencing products were purified using XTerminator^TM^ beads (Thermo Fisher Scientific, Waltham, USA) and automated sequencing performed by capillary electrophoresis on an ABI3500 (Applied Biosystems). Sequences were aligned and examined by visual inspection of the electropherogram (Fig. 2).

### MALDI MS assay design

Relevant mutations of *KRAS*, *NRAS* and *BRAF* have been identified from the COSMIC database and the respective literature. DNA sequences were extracted from the UCSC Genome Browser. These sequences were subsequently utilized to build our multiplex assay with Assay Design (v.3.0.0) covering *KRAS* mutations as rare as 0.002 % according to the COSMIC database (Table 1).

**Table 1:** Mutations covered in the MALDI MS array

KRAS: G12C, G12D, G12V, G12A, G12S, G12R, G13D,G13V, G13A, G13S, Q61H, Q61E, Q61K, Q61R, Q61P, Q61L, K117N, A146T
NRAS: G12C, G12D, G12V, G12A, G12S, G12R, G13D,G13V, G13A, Q61H, Q61E, Q61K, K117N, A146T
BRAF: V600E, V600K,V600R, V600L

### MALDI MS mutation detection

After semi-automated DNA-Isolation (QIAsymphony^®^, Qiagen, Hilden, Germany), DNA content was calculated by NanoDrop^TM^ spectrophotometry (Peqlab, Erlangen, Germany). PCR and downstream reactions were performed according to the iPLEX Pro Kit^®^ (Agena Bioscience, Hamburg, Germany) datasheet. In brief, a multiplex PCR-reaction (primers by Metabion, Munich, Germany) was performed at a final volume of 5 μl containing 10–100 ng template DNA leading to amplicon sizes ranging from 88 to 127 bp.

To dephosphorylate unincorporated dNTPs, 2 μL of a Shrimp-Alkaline-Phosphatase (SAP) Mix (iPlex^®^ Pro Kit) was added to each PCR reaction. After incubation steps according to the manufacturer’s protocol, an extension primer reaction was performed to hybridize and elongate the extension primers at the nucleotide position of interest.

**Fig. 3:**
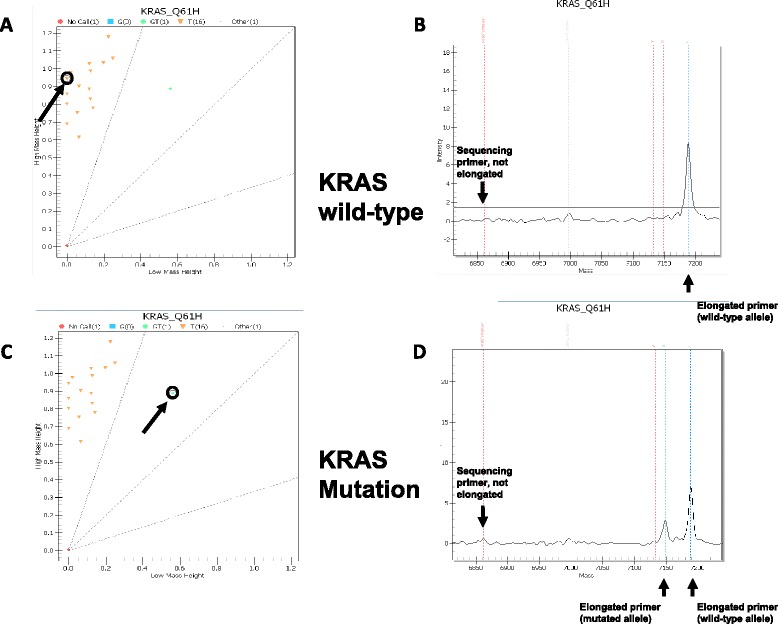
Representative examples of MALDI MS results. On the left, 16 cases plus wild type controle and no-template control tested for a *KRAS* mutation (p.Q61H). 15 cases are KRAS wild-type (including the wild-type control); one of the cases is highlighted with an arrow (**a**). In this specific case the sequencing primer (first arrow) is completely elongated by an Adenin indicated by a high peak in the mass range of the wild-type allele (second arrow) (**b**). One case shows a mutation in *KRAS* (c.183A > C, p.Q61H) (**c**). The reverse sequence-specific primer is completely consumed and elongated by either Thymine or Guanine (wild-type and mutated allele; arrows) (**d**)

Finally, the sample volume was increased by addition of 42 μl ultrapure water and free ions were removed by a resin cleanup step. 10–20 nl of the reaction products were dispensed onto a matrix-precoated 96- well SpectroCHIP^®^ bioarray by application of a nanodispenser (RS1000, Agena Bioscience). MS experiments were conducted on a MassArray^®^ Analyser 4 system according to the manufacturer’s protocol (Agena Bioscience, San Diego). This system is specifically designed for the detection of genetic mutations and not for the detection of other molecules. Results were analysed by MassArray^®^ Workstation software (v.3.3) (Agena Bioscience) (Fig. 3).

The general principle is based on amplification of the DNA by PCR, resulting in copies of both mutant and wildtype alleles. Primer extension performed using terminator nucleotides A, C, T, G, each with distinct masses leads to different masses of the amplicons depending on the mutational status that can subsequently be detected by mass spectrometry. If there is a mutant allele, three mass peaks may be seen: the unextended primer peak, the amplified wild-type allele peak and the amplified mutant allele peak (Fig. 3). The ratio of the areas under the curve of the wild-type allele and the mutant allele peaks are a quantitative measure of the percentage of mutant alleles.

### Next-generation sequencing (IonTorrent™)

For library preparation, the multiplex PCR-based Ion Torrent AmpliSeq™ technology (Thermo Fisher Scientific, Waltham, USA) was used. Amplicon library preparation was performed with the Ion AmpliSeq Library Kit v2.0 using 10 ng of DNA. Briefly, the DNA was mixed with the primer pool, containing all primers for generating the 180 amplicons and the Ampliseq HiFi Master Mix and transferred to a PCR cycler (BioRad, Munich, Germany). After the end of the PCR reaction, primer end sequences were partially digested using FuPa reagent, followed by the ligation of barcoded sequencing adapters (Ion Xpress Barcode Adapters 1–16, Thermo Fisher Scientific, Waltham, USA). The final library was purified using AMPure^®^ XP magnetic beads (Beckman Coulter, Krefeld, Germany) and quantified using qPCR (Ion Library Quantitation Kit, Thermo Fisher Scientific, Waltham, USA) on a StepOne^®^ qPCR machine (Thermo Fisher Scientific, Waltham, USA). The individual libraries were diluted to a final concentration of 100pM and eight to ten libraries were pooled and processed to library amplification on Ion Spheres using Ion PGM™ template OT2 200Kit. Unenriched libraries were quality-controlled using Ion Sphere quality control measurement on a QuBit^®^ instrument. After library enrichment (Ion OneTouch^®^ ES), the library was processed for sequencing using the Ion Torrent 200 bp sequencing v2 chemistry and loaded onto a chip. Data analyses were performed using the Ion Torrent Suite Software (v.4.2) as described previously. We use a custom designed colon cancer panel that includes *KRAS* and *NRAS* mutations in exon 2, 3 and 4.

## Results

### *KRAS* and *NRAS*

A total of 93 samples from patients with CRC were analysed by MALDI MS and the KRAS LCD-array Kit. Since the latter kit only detects *KRAS* mutations in exon 2, all cases with *KRAS* wild-type status were further analysed by Sanger sequencing of exons 3 and 4 of *KRAS* and exons 2,3 and 4 of NRAS (Fig. 1).

MALDI MS detected a *KRAS* mutation in 46/93 (49 %) tissue probes, from which 38/93 (41 %) were in exon 2, 3/93 (3 %) were in exon 3 and 5/93 (5 %) were in exon 4.

37/93 (40 %) of the *KRAS* mutations were already confirmed by the KRAS LCD-array kit, while additional 9/93 (10 %) of *KRAS* mutations were confirmed by subsequent Sanger sequencing.

A mutation in the *NRAS* gene was identified in 2/93 (2 %) of cases. MALDI MS and Sanger sequencing were in agreement in 2/2 (100 %) cases.

In total, MALDI MS results were in agreement to results obtained by the combination of the other two methods in 92/93 (99 %) cases.

### *RAS*-wild type tumors

Wild type tumors were analysed by three methods. Sanger sequencing identified 44/93 (47 %), the KRAS LCD-array kit 56/93 (60 %) and MALDI MS 44/93 (47 %) of patients with wild type tumors.

### One disconcordant case

In 1/93 (1 %) case a G12V mutation has been shown by MALDI MS and Sanger sequencing, but not by the KRAS LCD-array kit. In this single case, NGS had been performed and confirmed the presence of a G12V mutation with an allele frequency of 15 %. Thus, MALDI MS results were in agreement to results obtained by the combination of three methods in 93/93 (100 %) of the cases.

### *BRAF*

*BRAF* was mutated in 1/93 (1 %) case respectively. Again, MALDI MS and Sanger sequencing results were in perfect agreement (100 %).

All mutations found occurred exclusively. The results are summarized (Table 2).

**Table 2:** Detected mutations

		KRAS-LCD Array Kit	Sanger Sequencing	MALDI MS	NGS
KRAS					
Mutation		37	9	46	1
	A146T	0	4	4	0
	A146V	0	1	1	0
	G12A	2	0	2	0
	G12C	1	0	1	0
	G12D	16	0	16	0
	G12R	1	0	1	0
	G12S	1	0	1	0
	G12V	11	1	12	1
	G13D	5	0	5	0
	Q61H	0	3	3	0
Wild type		56	44	44	0
NRAS					
Mutation		2	0	2	0
	G12D	1	0	1	0
	G12V	1	0	1	0
Wild type		91	53	91	1
BRAF					
Mutation		0	1	1	0
	V600E	0	1	1	0
Wild type		93	52	92	1
Tested Samples		93	53	93	1

### Hands-on time and turnaround time

Overall hands-on time was shortest for the KRAS LCD-array Kit and MALDI MS (45 min), intermediate for NGS (70 min) and longest for Sanger sequencing (120 min).

The overall time-to-results was shortest for the KRAS LCD-array Kit (220 min), followed by MALDI MS (430 min), Sanger sequencing (715 min) and NGS (1020 min).

Approximated times to perform each step of the different methods namely KRAS LCD-array Kit, Sanger sequencing, MALDI MS and NGS are summarized (Table 3).

**Table 3:** Estimated Hands-on-time and Time-to-results

KRAS LCD-array Kit	Hands-on-time in minutes	Time-to-result in minutes
PCR reaction	15	150
Hybridization	20	60
Evaluation on PC	10	10
Overall	45	220
Sanger sequencing		
ᅟPCR reaction	15	150
ᅟGel preparation and electrophoresis	25	45
ᅟDucumentation and Evaluation of Dilution	10	10
ᅟEthanolprecipitation and centrifugation	20	35
ᅟElution of the Pellets in pure water	10	10
ᅟSequencing reaction	10	160
ᅟAdd purification beads and Incubation	10	40
ᅟSequencing run for all exons (forward and reverse)	10	250
ᅟEvaluation on PC	10	15
ᅟOverall	120	715
ᅟMALDI MS		
ᅟPCR reaction	15	150
ᅟDephosphorylation SAP reaction	5	60
ᅟCycled MassEXTEND reaction	10	150
ᅟSample conditioning, Nanodispensing	10	40
ᅟMS analysis and calling	5	30
ᅟOverall	45	430
ᅟNGS		
ᅟLibrary Preparation	30	300
ᅟEmulsion PCR	10	360
ᅟSequencing	20	300
ᅟData analysis	10	60
ᅟOverall	70	1020

## Discussion

According to recent clinical guidelines it is mandatory to evaluate the mutational status of oncogenes that act downstream of EGFR specifically *KRAS* and *NRAS* in mCRC in order to select a patient population that is most likely to benefit from anti-EGFR therapy [5, 8, 14, 25]. Additionally, genes such as *BRAF* that provide prognostic information may be included in the testing. Besides classical Sanger sequencing, new emerging techniques for the detection of genetic mutations are available. Among them MALDI MS has prompted particular interest among scientists and pathologists, since it combines high sensitivity and specificity with low cost per test, fast turnaround time and easy sample handling [17].

### Sensitivity and specificity

Sanger sequencing is generally considered to be the gold standard for the detection of mutations in *KRAS, NRAS* and *BRAF*. Specificity is generally high with all methods applied for the detection of genetic mutations [30, 32, 34–36]. However, sensitivity has been reported to differ [34, 37].

Whereas direct sequencing has been reproducibly shown to have a detection limit of >10 % mutant alleles, high resolution melting analysis has a lower detection limit of 10 % [38] that is similar to SnP shot assays (10 %) [27] and can further be improved by the cobasR test (5 %) [39], the TheraScreen^®^ test (1 %) [33, 38, 40–42] or Strip assays (1 %) [27, 38]. Also, NGS [34] and MALDI MS have similarly low detection limits of 1–5 % mutant allels [28, 33, 34, 38–44]. Altimari *et al.* [34] state that NGS was superior in terms of sensitivity and specificity compared to other techniques in detecting *KRAS* mutations in FFPE material, but MALDI MS was not included in their analysis.

To improve both, sensitivity and specificity, in direct sequencing, especially in specimen with low amount of DNA, it was recommended to increase the number of PCR cycles. Also to avoid false –positive errors, duplication of the test was expected to be effective [33]. Amplifiable DNA amounts are often limited when FFPE samples are used as a source, since DNA is highly fragmented by formalin treatment [33].

It has also been shown that various methods tested in different laboratories showed a decreasing correct mutational allele frequency proportionally with decreasing percentage of tumor cell content [26].

Therefore macro- or micro-dissection of the tumor area is usually done, which improves the test results and is therefore strongly recommended [45]. Tumor cell enrichment correlated significantly with the abundance of *KRAS*-mutated DNA [34].

Discordant results of different methods were attributed to tumor heterogeneity, contamination of the tumor sample with normal tissue, analytic factors affecting assay sensitivity and lack of experience with the respective method [19, 34, 35].

However, despite efforts to improve Sanger sequencing it has been shown that the sensitivity the specificity of mass spectrometrical methods exceeds that of traditional Sanger sequencing and is highly concordant with pyrosequencing, allelee-specific PCR [17, 46–48] and NGS [49]. In our study DNA quality was sufficient in all cases and none of the MALDI MS, NGS or Sanger sequencing reactions had to be repeated.

### Cost

When estimating the cost of a test, three parameters have to be considered, cost of instrumentation, consumable cost per test and hands-on-time. In the literature relatively few statements about costs of the different methods could be obtained.

Sarasqueta et al. reported low costs per test for direct sequencing and SNap shot^®^ compared to a Strip assay [27]. But the advantage of strip or chip hybridisation after PCR is low cost for technical equipment [45].

High performance of the PNA clamp PCR assay and low cost compared to TheraScreen^®^ test assay was reported by Norgard et al. [43]. The reagent costs for pyrosequencing and Sanger sequencing were comparable but higher than that of melting curve analysis in one study [30].

Likewise, costs per sample in Sanger sequencing is higher compared to MALDI MS, especially when complex testing of numerous mutations was performed [17]. In a previous study, we reported equipment cost to be highest with Sanger and pyrosequencing, followed by real-time- and array-based systems. Costs per sample were lowest for Sanger and pyrosequencing, two-fold higher for the array and three-fold higher for high resolution melting curve analysis [45].

NGS and MALDI MS have comparable costs for the technical equipment, but costs per test are much lower in MALDI MS especially when a customized assay for mutation detection is build. It is important to mention, that local prices for equipment and tests may vary considerably.

Hands-on-time is an important cost factor and has been reported to be around 2 h for Sanger sequencing, SNap shot^®^ assays, Therascreen, high resolution melting curve analysis, NGS and MALDI MS [27, 38, 50]. The StripAssays may be conducted within 1,5 h [27]. Melting curve analysis has the shortest hands on time compared to the other mentioned methods [30, 45]. In our study hands-on time was around 45 min for performing the KRAS LCD-Kit and MALDI MS, 70 min for NGS and around 2 h for Sanger sequencing.

In the authors opinion MALDI MS is the most cost effective method to detect clinically relevant mutations in CRC. Since it is an open platform more mutational hotspots for testing may be easily added. However, this holds only true for high throughput laboratories, since the equipment costs are rather high compared to array- or real-time PCR based methods or high resolution melting curve analysis. For laboratories with low throughput, techniques with low equipment costs and high costs per sample may be more cost effective on the short and also on the long term. Therefore definite conclusions regarding cost-effectiveness cannot easily be generalized.

### Turnaround time

Besides hands-on-time turnaround time/time-to-result is important and has been reported to be 2 working days for Sanger sequencing, 1.5 working days for SNaP shot and pyrosequencing and 1 working day for Strip- and chip assays [27, 38, 45, 51] which is in line with our results. With respect to turnaround time high resolution melting curve analysis has been reported to outperform the former mentioned methods [45]. We perform MALDI MS mutational testing within 1.5 working days. In our opinion a time-to-result of around 2 working days seems reasonable, but if the time line is critical one might choose high resolution melting curve analysis as the preferred method. However, as previously mentioned this method harbours some disadvantages as false-positive results may occur more frequently and costs per test are high compared to the other methods [38, 45].

### KRAS and NRAS

We detected KRAS mutations by MALDI MS in 49 % of all samples analysed which is in perfect accordance with other reports [17, 41, 45]. In one case MALDI MS and Sanger sequencing detected a mutation but the KRAS LCD-array kit failed to do so. At first, it was unclear whether the detection of the G12V mutation could be attributed to lower detection limits of MALDI MS compared to the chip-assay or if this case represented a false-positive result. Since it has been proven in the past that MALDI MS and NGS yield similar results [49], a NGS experiment has been performed. Indeed, the very same mutation could be confirmed at an allelic frequency of approximately 15 % by NGS. Taken all three alternative methods together, MALDI MS showed concordant results in 100 % of the cases. This proves the perfect reliability of MALDI MS mutational testing. It is recommended by several authors to test for all codons of KRAS. We included mutations in our panel that occurred in frequencies as low as 0.002 % of cases in CRC according to the COSMIC database because in our opinion this represents a reasonable trade-off between sensitivity and wise handling of resources. NRAS mutations were detected in 3 % of our cases, again well in accordance with other reports [8, 10, 11]. Concordance of NRAS testing was 100 % between the three methods applied. As all KRAS and NRAS mutations have been shown to have lower response rates to anti-EGFR therapy compared to RAS wild-types [10], mutational testing for both should be standard of care for all mCRC.

### BRAF

Besides mutations of *KRAS* and *NRAS*, the *BRAF* gene plays a critical role in CRC. It has been shown that *BRAF* mutations are frequent in sporadic CRC with MSI (32–74 %) and in serrated polyps (up to 90 % in sessile serrated adenomas [52]). The improved classification of serrated lesions by *BRAF* mutation testing may bet the key to identify lesions with a higher potential to progression into BRAF V600E mutated CRC [53].

This subset of tumors is characterized by right-sided location in the colon, prevalence of mucin, high levels of promoter methylation of CpG islands (CIMP) and a good prognosis compared to its *BRAF* wild type counterparts with 5-year survival rates over 70 % [17, 54, 55]. In contrast a small subset of *BRAF* mutated CRC harbour MSS (4 %) and have a significantly worse prognosis with 5-year survival rates of only 16,7 % [21]. Testing for a *BRAF* mutation alone is therefore not sufficient but adds significant prognostic information in combination with MSI/MSS testing. Of note is that hereditary non-poliposis colorectal carcinomas (HNPCC) generally do not exhibit BRAF mutations, therefore it might be tested to exclude such a hereditary form of CRC.

Thus we added testing for *BRAF* mutations to our customized panel in order to be able to provide improved prognostic information in conjunction with microsatellite testing.

In our cohort one case with *BRAF* mutation has been found by MALDI-MS and Sanger Sequencing. Our incidence of only 1 % *BRAF* mutations is probably due to the rather low sample size considering that *BRAF* mutations have been reported in the literature at a frequency of 5–20 % of all CRC.

### MALDI MS

The MALDI MS technology has the advantage that tests with adequate quality standards may be designed and that it is an open platform which allows fast inclusion of complex mutations of various gens that may be important in the future. Although the clinical significance of mutations with low allelic frequency in relation to prognosis and therapeutic benefit has yet not fully understood, MALDI MS is very specific, significantly more sensitive than Sanger sequencing and reaches detection limits comparable to other modern technologies such as NGS. In addition, for laboratories with a high throughput it combines the advantage of low hands-on time, fast turnaround time and cost effectiveness.

## Conclusion

Taken together evaluation of KRAS and NRAS mutational status for therapeutic requirements and BRAF mutational analysis by MALDI MS for prognostic and classification purposes is clearly an attractive approach for routine diagnostics.

## Abbreviations

ARMS: Amplification refractory mutation system
ASCO: American Society of Clinical Oncology
Bp: Basepair
BRAF: V-Raf murine sarcoma viral oncogene homolog B
CRC: Colorectal cancer
COSMIC: Catalogue Of Somatic Mutations In Cancer
mCRC: Metastasized Colorectal Cancer
DNA: Deoxyribonucleic acid
EGFR: Epidermal growth factor receptor
ESMO: European Society for Medical Oncology
H&E: Hematoxylin and Eosin
HNPCC: Hereditary non-poliposis colorectal carcinomas
KRAS: V-Ki-ras2 Kirsten rat sarcoma viral oncogene homolog
MALDI MS: Matrix assisted laser desorption/ionization mass spectrometry
MSI: Microsatellite instable
MSS: Microsatellite stable
NGS: Next-Generation Sequencing
NRAS: Neuroblastoma RAS viral oncogene homolog
NTP: Nucleoside triphosphate
PCR: Polymerase chain reaction
PIK3CA: Phosphatidylinositol-4,5-bisphosphate 3-kinase, catalytic subunit
PTEN: Phosphatase and tensin homolog
RAF: Rapidly Accelerated Fibrosarcoma (protein family)
RAS: Rat sarcoma (protein family)
SAP: Shrimp-Alkaline-Phosphatase
UCSC: University of California, Santa Cruz
WSC: Wildtype Supressor Compound
